# The Dynamics of Naturally Acquired Immune Responses to *Plasmodium falciparum* Sexual Stage Antigens Pfs230 & Pfs48/45 in a Low Endemic Area in Tanzania

**DOI:** 10.1371/journal.pone.0014114

**Published:** 2010-11-29

**Authors:** Teun Bousema, Will Roeffen, Hinta Meijerink, Harry Mwerinde, Steve Mwakalinga, Geert-Jan van Gemert, Marga van de Vegte-Bolmer, Frank Mosha, Geoffrey Targett, Eleanor M. Riley, Robert Sauerwein, Chris Drakeley

**Affiliations:** 1 Department of Immunology & Infection, Faculty of Infectious & Tropical Diseases, London School of Hygiene & Tropical Medicine, London, United Kingdom; 2 Joint Malaria Programme, Moshi, Tanzania; 3 Department of Medical Microbiology, Radboud University Nijmegen Medical Centre, Nijmegen, The Netherlands; 4 TPC District Designated Hospital, Tanzania Plantation Company, Lower Moshi, Tanzania; 5 Department of International Health, Immunology, and Microbiology, University of Copenhagen, Copenhagen, Denmark; 6 Kilimanjaro Clinical Research Institute, Moshi, Tanzania; Menzies School of Health Research, Australia

## Abstract

**Background:**

Naturally acquired immune responses against sexual stages of *P. falciparum* can reduce the transmission of malaria from humans to mosquitoes. These antigens are candidate transmission-blocking vaccines but little is known about the acquisition of sexual stage immunity after exposure to gametocytes, or their longevity and functionality. We conducted a longitudinal study on functional sexual stage immune responses.

**Methodology/Principal Findings:**

Parasitaemic individuals (n = 116) were recruited at a health centre in Lower Moshi, Tanzania. Patients presented with gametocytes (n = 16), developed circulating gametocytes by day 7 (n = 69) or between day 7 and 14 (n = 10) after treatment or did not develop gametocytes (n = 21). Serum samples were collected on the first day of gametocytaemia and 28 and 84 days post-enrolment (or d7, 28, 84 after enrolment from gametocyte-negative individuals). Antibody responses to sexual stage antigens Pfs230 and Pfs48/45 were detected in 20.7% (72/348) and 15.2% (53/348) of the samples, respectively, and were less prevalent than antibodies against asexual stage antigens MSP-1_19_ (48.1%; 137/285) and AMA-1 (52.4%; 129/246)(p<0.001). The prevalence of anti-Pfs230 (p = 0.026) and anti-Pfs48/45 antibodies (p = 0.017) increased with longer duration of gametocyte exposure and had an estimated half-life of approximately 3 months. Membrane feeding experiments demonstrated a strong association between the prevalence and concentration of Pfs230 and Pfs48/45 antibodies and transmission reducing activity (TRA, p<0.01).

**Conclusions/Significance:**

In a longitudinal study, anti-Pfs230 and Pf48/45 antibodies developed rapidly after exposure to gametocytes and were strongly associated with transmission-reducing activity. Our data indicate that the extent of antigen exposure is important in eliciting functional transmission-reducing immune responses.

## Introduction

Recent successes in eliciting transmission-reducing immune responses with malaria transmission-blocking vaccines (MTBV) in animal models [1], [2], [3], [4] have fuelled interest in future deployment of these vaccines as part of malaria control and elimination strategies [5], [6]. Understanding the dynamics of naturally acquired transmission reducing immune responses will assist in the future deployment of MTBV in endemic populations. The transmission of malaria depends on the presence of mature sexual stage parasites, gametocytes, in the human peripheral blood. Once ingested by a mosquito taking a blood meal, gametocytes will form male microgametes and female macrogametes that after fertilization and zygote formation develop into ookinetes that penetrate the mosquito midgut wall to form an oocyst underneath the basal lamina of the midgut. Each oocyst produces thousands of sporozoites, rendering the mosquito infectious to humans. The infectiousness of gametocytes to mosquitoes depends on their density [7], [8], [9] and level of maturation [10] but also on mosquito [11] and human immune responses [12]. Human transmission-reducing antibodies are ingested by Anopheline mosquitoes together with gametocytes and can interfere with zygote formation and, as a consequence, further development of parasites in the mosquito vector. MTBV are designed to induce such antibody responses to reduce the infectiousness of gametocytes to mosquitoes or even block transmission completely [6], [13], [14].

The three most promising MTBV candidates for *Plasmodium falciparum* so far are based on the induction of antibody responses against parasite antigens Pfs230, Pfs48/45 and Pfs25 [1], [2], [3], [4], [15]. Pfs48/45 and Pfs230 are expressed on the surface of gametes and are involved in the fertilization of macrogametocytes by microgametes [5]; Pfs25 is a postfertilisation antigen expressed on the surface of ookinetes [4], and plays a role in the traversal of the mosquito midgut epithelium [16]. The gamete surface molecules Pfs48/45 and Pfs230 are also expressed in gametocytes circulating in the human blood and antibody responses against these antigens are detected in naturally exposed individuals [12], [17], [18]. This makes it possible to study the nature and duration of sexual-stage specific immunity in naturally infected individuals [5]. Naturally acquired sexual stage-specific antibody responses may be acquired after exposure to gametocytes [12] and rapidly induced as part of the initial response to infection [19], [20]. In contrast to antibodies against pre-erythrocytic and blood-stage antigens [19], [21], [22], the prevalence of sexual-stage specific antibodies does not increase with age [23], [24]. Little is known about the rate of induction or longevity of sexual-stage antibody responses and this may be important for natural boosting of any vaccine induced response.

Here, we determine the acquisition, longevity and functionality of sexual stage specific antibody responses in a longitudinal study in an area of low endemicity in Tanzania.

## Methods

Ethics permission was received from the ethical committees of the National Institute for Medical Research, the Kilimanjaro Christian Medical Centre, and the London School of Hygiene and Tropical Medicine. Written consent was obtained from all participants or their parents/guardians prior to enrolment in the study.

Individuals were recruited from January 2003 till December 2004 at the health centre in Msitu wa Tembo and the clinic of the nearby sugar plantation, Tanzania Plantation Company, in Lower Moshi (latitude 3° 33′ S; longitude 37° 17′ E). The area of Lower Moshi lies at an altitude of ∼700 meters between the Maasai savannah and foothills of Mount Kilimanjaro. Malaria transmission at the time the study was conducted was hypoendemic with an estimated entomologic inoculation rate of 3.4 (95% CI 0.7–9.9) infectious bites per person per year [25] and peaks in malaria incidence following the rains in March–May and October–November. The low level of malaria transmission was considered to be beneficial for the current study objectives because of the low likelihood of re-infections during follow-up. Individuals were eligible for recruitment if they were found parasitaemic after examination of 100 high power microscopic fields. Slides were read for asexual parasites and gametocytes separately, each by two technicians. Asexual parasites and gametocytes were enumerated against 200 and 500 leukocytes, respectively, and expressed as density/µL assuming an average leukocyte count of 8,000/µL. Parasitaemic individuals were treated with sulphadoxine-pyrimethamine (SP) according to national guidelines at that time and seen on day 7, 14, 28, 42, 56 and 84 after initiation of treatment. On days 7, 14, 28, and 84, a slide was taken to determine exposure to asexual parasites and gametocytes; a rapid diagnostic test [Paracheck®, Orchid Biomedical Systems, India] was used to detect infection qualitatively on day 42 and 56. On the first day of gametocytaemia and on days 28 and 84 after enrolment, a venous blood sample was taken for serological analyses and standard membrane feeding assay. Samples were also taken from individuals who did not present with microscopically detectable gametocytes in the 28 days post treatment. For these individuals, sampling was done on day 7, 28 and 84 after enrolment. This observational study was exploratory in nature and no formal sample size calculation was done.

### Enzyme-linked immunosorbent assays (ELISAs)

#### Antigen preparation

Mature gametocytes of *P. falciparum* (NF54 strain) were produced in an automated static culture system [26], isolated on 63% Percoll [no. 17-0891-01, GE Healthcare, Uppsala, Sweden] and stored at −70°C until used. The NF54 gametocytes were extracted in 25 mM Tris-HCl, pH 8.0, supplemented with 150 mM NaCl, 1.0% sodium desoxycholate, and 1 mM phenylmethylsulfonyl fluoride. Samples were centrifuged at 13,000×g for 5 minutes at room temperature to remove insoluble debris and the supernatant was used as the source of whole parasite antigen.

#### ELISA for sexual stage antigens Pfs230 and Pfs48/45

The presence of human antibodies to Pfs48/45 and Pfs230 was determined by a two-site ELISA as described previously [19]. Briefly, 96-well microtiter plates (Sterilin-ELISA plates [no. 53011; International Medical Products B.V., Zutphen, The Netherlands] were coated with anti-Pfs230 mouse monoclonal antibody (MAb) 63F2A2 (5 µg/mL) in phosphate-buffered saline (PBS), pH 7.4. For Pfs48/45, the plates were coated with rat MAb 85RF45.3. Free sites were blocked with 5% low-fat dry milk [Marvel, Premier International Foods Ltd., Spalding, United Kingdom] in PBS. Pfs48/45 and Pfs230 were captured from a gametocyte extract (250,000 parasite equivalents/well) for half of the wells; the other half was tested without antigen. Serum samples (1∶ 100 dilution) were added to the wells and incubated for two hours. Bound IgG antibodies were detected by horseradish peroxidase–labeled goat anti-human IgG [31412; Pierce Biotechnology, Inc., Rockford, IL]. The wells were washed three times with PBS and subsequently incubated with tetramethylbenzidine substrate (TMB) solution for 20 minutes. The colour reaction was stopped with 2 N H2SO4, and optical density (OD) was read at 450 nm [Anthos 2001 microplate reader; Labtec BV, Heerhugowaard, The Netherlands]. All incubations were carried out at room temperature. All serum samples were tested in duplicate with a concurrent positive control and a minimum of four negative (Dutch blood bank donor) controls per plate. The OD was adjusted for background reactivity by subtracting the OD without antigen.

#### ELISA for asexual stage antigens Apical Membrane Antigen-1 (AMA-1) and Merozoite Surface Protein-1_19_ (MSP-1_19_)

Anti-MSP-1_19_ and anti-AMA-1 human IgG antibodies were detected by ELISA using standard methodology [21], [27], using the sera at 1/1000 and 1/2000 dilution, respectively. The OD was standardized against a positive control that was included on each plate.

For sexual and asexual stage ELISAs, all samples from one individual were included on the same plate; a cut-off above which samples were deemed antibody positive was defined using a mixture model as previously described [27]. The distribution of OD values was subsequently fitted as the sum of two Gaussian distributions (a narrow distribution of sero-negatives and a broader distribution of sero-positives) using maximum likelihood methods. The mean OD of the Gaussian corresponding to the sero-negative population plus three standard deviations was used as the cut-off for sero-positivity.

### Standard membrane feeding assay (SMFA)

Twenty-five individuals were selected for membrane feeding experiments. The selection was done randomly; the only selection criterion was the availability of a complete set of three serum samples from the first day of patent gametocytaemia, day 28 and day 84after enrolment. Seventy-five serum samples from these 25 individuals were purified for the SMFA using the Protein G HP Multitrap [GE Healthcare, Uppsala, Sweden: Code no 28-9031-35] according to the manufacturer's instructions. 200µL plasma was diluted with 400µl binding buffer and added in triplicate to the 96-well plate. The flow through (FT) still contains antibody and can be isolated using the same procedure twice. The eluted and neutralized samples were combined, diluted to 20ml with MilliQ water and subsequently concentrated to the original starting volume using centrifugal concentration tubes [Vivaspin 20, Sartorius AG, Goettingen, Germany: Code no VS2021]. The purified field samples were used for testing in the ELISAs, or added to freeze dried FCS (90µl) tubes for testing in the membrane feeding assay. The yield of protein varied from 2 to 4 mg/ml and a mean recovery of 76.2% (standard deviation of 13.9%) varied between 51% and 97%.

Experimental infections of mosquitoes were carried out as previously described [28], [29] including samples from the same individual in the same experiment. Three to five day old *A. stephensi* were allowed to feed on cultured *P. falciparum* gametocytes of NF54, in the presence of purified control serum or test sample at a final dilution of 1∶3 in the feeder. After feeding, blood-fed mosquitoes were kept at 26°C. Surviving mosquitoes were dissected seven days later, and oocyst counts were made for each sample. Midguts of 20 mosquitoes were examined for oocysts. The experiment was considered valid when at least 70% of the mosquitoes feeding on control sera were infected. The observed transmission reducing activity (TRA) of serum was determined as the percentage reduction in arithmetic mean oocyst number in test samples as compared to paired controls [30].

### Statistical analysis

Data was double entered in MS Access and imported into STATA version 11 (StataCorp LP, College Station, TX, USA). Binary variables were analysed by Chi-square, Fisher's Exact or Chi-square test for trend; odds ratio's with 95% confidence intervals were presented where appropriate. The association between continuous variables was determined by the nonparametric Spearman correlation coefficient. To determine the longevity of the antimalarial antibody responses, we analysed the change in concentrations of antibodies to Pf230 and Pfs48/45 between day 28 and day 84 in relation to time (in days) since the last documented exposure to gametocytes (day 0–28). The longevity of antibody responses to MSP-1_19_ and AMA-1 was estimated in a similar manner, using the change in antibody concentration between day 7 and day 84 in relation to time since the last documented exposure to asexual parasites (day 0). The half-life of the antibody response was analysed in a repeated measurements analysis using log-linear regression model including adjusting for associations between multiple data points from the same subjects. Half-lives were calculated from the estimated decay rate and the boundaries at 95% confidence interval obtained from the mixed-effects model [31]. A curve was fitted for Pfs230 and Pfs48/45 antibody concentration in relation to the reduction in oocyst numbers using GraphPad Prism software 4.02 (GraphPad Software, Inc., CA, USA).

## Results

**Figure 1:**
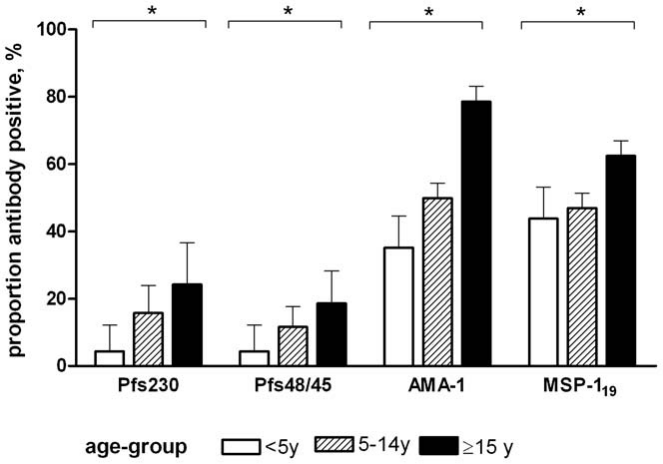
Antibody responses to Pfs230 and Pfs48/45 in relation to age. Bars indicate antibody prevalence in different age-groups, error bars the upper limit of the 95% confidence interval. Multiple observations are included per individual. Antibody responses to Pfs230 (p = 0.006), Pfs48/45 (p = 0.008), AMA-1 (p<0.001) and MSP-1_19_ (p = 0.013) increased with increasing age, after adjusting for correlations between observations from the same individual. The number of observations for Pfs230, Pfs48/45, AMA-1 and MSP-1_19_ were 69, 69, 31, 32 (<5 years), 168, 168, 134, 134 (5–14 years), 111, 111, 119, 81 (≥15 years). The asterisks indicates a statistically significant positive trend between antibody responses and age in all categories (p<0.05).

Sexual stage antibody responses were examined in 116 individuals who presented with asexual *P. falciparum* parasites at the clinic and who had gametocytes at enrolment (n = 16), developed gametocytes on day 7 (n = 69) or day 14 (n = 10) after treatment with SP or did not develop on any day gametocytes after treatment (n = 21) (table 1). After clearance of the initial infection, no re-infections were detected during follow-up by microscopic slide (day 7, 14, 28, 84) or by rapid diagnostic test (day 42, 56). Anti-Pfs230 antibodies were detected in 20.7% of the samples (72/348); 25.9% (30/116) of the participants showed Pfs230 seroreactivity on at least one time-point during the study. Antibodies against Pfs48/45 were detected in 15.2% (53/348) of the samples; 27.6% (32/116) of the participants showed Pfs48/45 seroreactivity on at least one occasion. There was a strong positive association between the prevalence of gametocytes (p<0.001) and concentration of antibodies against Pfs230 and Pfs48/45 (Spearman correlation coefficient 0.66, p<0.001). Recognition of Pfs230 and Pfs48/45 increased with age in our population (figure 1). Antibody responses to asexual stage antigens AMA-1 and MSP-1_19_ were detected in 52.4% (129/246) and 48.1% (137/285) of the samples and antibody prevalence increased with age (figure 1).

**Table 1:** Demographic information.

Age, median (IQR)	10 (5–18)
Gender, % female	42.1 (48/114)
Asexual parasite density/µL, median (IQR)	133 (34–812)
Proportion presenting with fever (≥37.5°C), %(n/N)	56.6 (64/113)
Clinical malaria (parasite density>1000/µL & fever)	11.5% (13/113)
First day of gametocytaemia	
Never	18.1 (21/116)
Prior to treatment	13.8 (16/116)
7 days post initiation treatment	59.5 (69/116)
14 days post initiation treatment	8.6 (10/116)
28–84 days post initiation treatment	0.0 (0/116)
Duration of gametocytaemia	
<2 weeks	75.0 (69/92)
2–3 weeks	16.3 (15/92)
>3 weeks	8.7 (8/92)

### The induction and duration of sexual stage antibody responses

**Figure 2:**
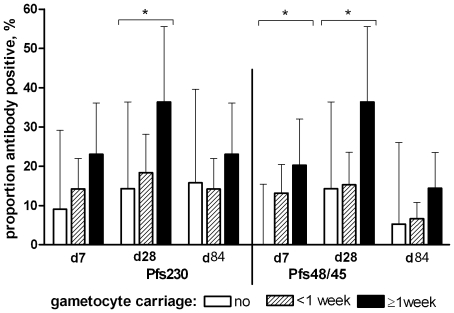
Pfs230 and Pfs48/45 antibody prevalence in relation to the duration of exposure to gametocytes during follow-up. Bars indicate total antibody prevalence in individuals who did not develop gametocytes during follow-up (clear bars), individuals who had gametocytes detected by microscopy on only one occasion (shaded bars, less than one week) and individuals who had gametocytes detected on at least two consecutive visits that were at least 7 days apart (filled bars, ≥1 week); error bars indicate the upper limit of the 95% confidence interval. On day 7 and 28 after enrolment, the number of observations from individuals without gametocyte exposure was 22, from individuals with gametocytes <1 week 69, and from individuals with gametocytes for ≥1 week 25. On day 84, these figures were 19, 69 and 25. The asterisks indicates a statistically significant positive trend between antibody responses and duration of exposure to gametocytes (p<0.05).

Sexual stage antibody prevalence and concentration were related to recent cumulative exposure to gametocytes prior to sampling. There was no statistically significant association between the duration of gametocyte exposure and Pfs230 antibody prevalence on day 7 after enrolment (OR 1.74, 95% CI 0.79–3.87, p = 0.17). Pfs230 antibodies on day 28 after enrolment were less prevalent in individuals who had no microscopically detectable gametocytes between day 0 and day 28, more prevalent in those who had gametocytes on only one occasion during this period (<1 week) and most prevalent in individuals who had gametocytes for ≥1 week (trend: OR 2.44, 95% CI 1.11–5.36; p = 0.026). A similar association was observed for Pfs48/45 antibodies on day 7 (trend: OR 2.62, 95% CI 0.98–7.01, p = 0.054) and on day 28 (OR 2.73, 95% CI 1.19–6.35; p = 0.017) after adjustment for age (figure 2; table 2). Similarly, an increase in day 28 antibody concentrations (optical density in the ELISA) for Pfs230 (β = 0.12, 95% CI 0.01–0.23, p = 0.046) and Pfs48/45 (β = 0.060, 95% CI 0.00–0.12, p = 0.066) was observed across these gametocyte carriage categories, after adjustment for age (table 2). These trends disappeared by day 84 for Pfs230 antibody prevalence (OR 1.29, 95% CI 0.52–3.20, p = 0.58) and concentration (β = 0.051, 95% CI -0.03–0.14, p = 0.24) and for Pfs48/45 antibody prevalence (OR 1.93, 95% CI 0.46–8.14, p = 0.37) and concentration (β = 0.039, 95% CI 0.00–0.08, p = 0.074). Although sexual stage antibody prevalence was least common in those who did not develop gametocytes during follow-up (p<0.001), there was a borderline significant increase in the prevalence of both Pfs230 (p = 0.076) and Pfs48/45 antibodies (p = 0.14) between day 7, 28, and 84 in this group. There was no association between the duration of gametocyte exposure and the antibody prevalence or concentration for AMA-1 (p≥0.42) and MSP-1_19_ (p≥0.48) on any of the days of sampling.

**Table 2:** Pfs230 and Pfs48/45 antibody prevalence and concentration in relation to the duration of exposure to gametocytes during follow-up.

		Pfs230		Pfs48/45	
Sampling time-point	Gametocyte exposure during follow-up	Antibody prevalence, % (n/N)	Antibody concentration, median OD (IQR)	Antibody prevalence, % (n/N)	Antibody concentration, median OD (IQR)
Day 7	No	9.1 (2/20)	0.09 (0.03–0.14)	0.0 (0/22)	0.07 (0.04–0.15)
	<1 week	15.9 (11/69)	0.11 (0.04–0.18)	14.7 (10/69)	0.10 (0.04–0.19)
	≥1 week	24.0 (6/25)	0.18 (0.08–0.25)	20.0 (5/25)	0.14 (0.06–0.29)
Day 28	No	14.3 (3/21)*	0.15 (0.05–0.18)*	14.3 (3/21)*	0.09 (0.08–0.20)¥
	<1 week	21.7 (15/69)*	0.11 (0.04–0.20)*	17.4 (12/69)*	0.10(0.06–0.20)¥
	≥1 week	44.0 (11/25)*	0.28 (0.11–0.50)*	44.0 (11/25)*	0.25(0.08–0.49)¥
Day 84	No	15.8 (3/19)	0.08 (0.01–0.22)	5.3 (1/19)	0.08 (0.02–0.16)¥
	<1 week	15.9 (11/69)	0.08 (0.03–0.18)	5.8 (4/69)	0.07(0.02–0.16)¥
	≥1 week	24.0 (6/25)	0.16 (0.07–0.29)	12.0 (3/25)	0.16(0.04–0.27)¥

Antibody concentration (optical density, OD, in the ELISA) was adjusted for background reactivity by subtracting the OD without antigen; seropositive and seronegative individuals are included in the table.

* Trend-test, p<0.05;

¥ 0.05>p<0.1.

The decay of Pfs230 and Pfs48/45 antibody responses was determined in individuals who had serum samples collected on day 28 and day 84 and who were antibody positive on day 28 without evidence for continued exposure to gametocytes or re-infection with asexual parasites after day 28. For Pfs230 antibodies, 60 paired observations were available for 30 individuals and the half-life of antibody responses was estimated at 92.6 (95% CI 74.7–122.0) days. This half-life of Pfs230 antibodies appeared shorter in children below 10 years of age (73.9 days, 95% CI 58.6–99.9) compared to adults (100.5 days, 95% CI 76.2–147.9) although not statistically significant (p = 0.10). For Pfs48/45 52 paired observations were available from 26 individuals and the half-life of antibody responses was estimated at 83.0 (65.8–112.3) days. Similarly for Pfs48/45, the half-life of antibody responses appeared shorter in children below 10 years of age (70.2 days, 95% CI 54.1–100.0) compared to older individuals (90.3 days, 95% CI 65.7–144.0) although not statistically significant (p = 0.25). The half-lives for Pfs230 and Pfs48/45 antibody responses were similar to those of responses against AMA-1 (97.7 days, 95% CI 78.6–129.1; based on 140 observations from 52 individuals) and MSP-1_19_ (52.4 days, 95% CI 44.5–63.6; 145 observations from 52 individuals). AMA-1 and MSP-119 antibody responses declined gradually after enrolment (p≤0.001).

### The functionality of sexual stage antibody responses

Seventy five samples from 25 individuals were included in the SMFA (table 3), giving an overall median reduction of oocyst numbers of 19.4% (IQR 0–66.7). Of the 25 individuals who were included in the SMFA, 6 (24%) were consistent responders to Pfs230 (i.e. Pfs230 antibody positive on all three days), 3 (12%) consistent responders to Pfs48/45 and 3 (12%) consistently reduced transmission ≥50% (TRA≥50, table 3). Individuals who showed TRA≥50% on all three occasions were significantly more likely to be a consistent responder to Pfs48/45 (p = 0.002) and Pfs230 (p = 0.065), although the latter was not statistically significant.

**Table 3:** Individual data from repeated membrane feeding experiments.

ID	Age	First-last day gametocytes	Pfs230 antibody prevalenced7-d28-d84	Pfs48/45 antibody prevalenced7-d28-d84	TRA≥50% and ≥90% prevalenced7-d28-d84
468	32	D0	0-0-0	0-0-0	0-0-0
565	8	D7	0-0-0	0-0-0	0-0-0
842	5	D7	0-0-0	0-0-0	0-0-0
849	10	D7	0-0-0	0-0-0	0-0-0
603	25	D14	0-0-0	0-0-0	0-0-0
649	13	D14	0-1-0	0-0-0	0-0-0
585	5	D7	0-0-0	1-1-0	0-0-0
639	29	D0–28	1-1-1	1-1-0	0-0-0
655	9	D7	1-1-0	1-1-0	0-0-0
724	11	Not detected	1-1-1	0-0-0	0-0-0
554	8	D7	0-0-0	0-0-0	1-0-0
527	8	D7	0-1-1	0-0-0	0-1-0
829	41	D7	0-1-1	1-1-1	0-1-0
886	12	D14–28	1-1-1	0-1-0	0-1-0
910	5	D7	1-1-0	0-0-0	0-1-0
752	10	Not detected	1-1-1	0-0-0	0-1-0
754	12	Not detected	0-0-0	0-1-0	0-1-0
1045	29	Not detected	0-0-0	0-0-1	0-0-1
1049	16	D7–28	0-1-1	0-1-0	0-2-0
579	6	D7	0-0-0	0-1-0	1-1-0
801	12	D0	1-1-0	1-1-0	2-1-0
902	10	D7	0-1-1	0-1-0	0-2-2
596	6	D0–28	0-1-1	0-1-1	1-2-2
889	46	D7	1-1-1	1-1-1	2-2-2
826	41	D14	1-1-1	1-1-1	2-2-2

Data are given for 25 individuals who had samples taken on three time-points: day 7 (d7), day 28 (d28) and day 84 (d84). For Pfs230 and Pfs48/45 seroreactivity: 0 = absence of antibodies; 1 = presence of antibodies. For Transmission Reducing Activity (TRA) in the standard membrane feeding assay: 0 = <50% reduction in the number of oocysts; 1≥50% reduction; 2≥90% reduction. Individuals are ranked based on their TRA in the standard membrane feeding assay.

**Figure 3:**
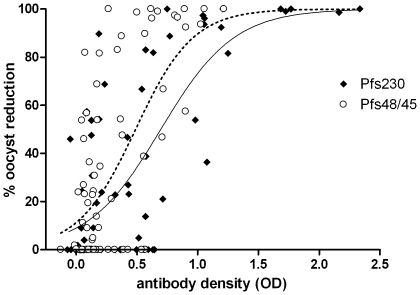
The relation between transmission reducing activity and Pfs230 and Pfs48/45 antibody concentration. Transmission reducing activity is plotted as a continuous variable, the reduction in oocyst density in mosquitoes, on the Y axis against antibody concentration (optical density, OD) on the X-axis. Filled diamonds represent Pfs230 antibody responses; open circles Pfs48/45 antibody responses. The solid line represents the best fit for the association between Pfs230 antibody concentration and % oocyst reduction; % oocyst reduction = 100/1+10^((0.697-OD Pfs230)*1.44)^; R^2^ = 0.56. The dashed line the association between Pfs48/45 antibody concentration and % oocyst reduction: % oocyst reduction = 100/1+10^((0.490-OD Pfs48/45)*1.84)^; R^2^ = 0.45.

When samples were considered individually (n = 75), TRA≥50% was significantly associated with Pfs230 antibody prevalence (OR 13.14; 95% CI 1.90–91.16, p = 0.009 after adjustment for repeated measures) and Pfs48/45 antibody prevalence (OR 43.04; 95% CI 3.21–577.44, p = 0.005). Twelve samples, derived from six individuals, reduced oocyst numbers ≥90% (TRA≥90%), 100% (12/12) of these were positive for Pfs230 antibodies and 91.7% (11/12) for Pfs48/45 antibodies. We observed a strong positive association between the reduction in oocyst numbers in the SMFA and Pfs230 (Spearman correlation coefficient = 0.65, p<0.001) and Pfs48/45 antibody concentration (Spearman correlation coefficient = 0.54, p<0.001). Samples with a higher antibody concentration were more likely to reduce oocyst numbers (figure 3). In a multivariate model that incorporated reactivity to both antigens, there was a strong association between the reduction in oocyst numbers and Pfs230 (β = 35.93, 95% CI 22.35–49.51, p<0.001) and Pfs48/45 antibody concentration (β = 44.57, 95% CI 20.49–68.85, p<0.001). Of all samples with an OD>1 in the Pfs230 ELISA, 83.3% (10/12) showed TRA≥90%; 87.5% of the samples with an OD>0.8 in the Pfs48/45 ELISA showed TRA≥90%. All samples (6/6) that fulfilled both these criteria reduced oocyst numbers by ≥90%.

## Discussion

In this study, we determined the acquisition, longevity and effectiveness of antibody responses against *P. falciparum* sexual stage antigens Pfs230 and Pfs48/45 in individuals residing in an area of low endemicity in Tanzania after a recent malaria episode. Antibody prevalence and concentration were related to the duration of gametocyte exposure and showed a strong association with transmission- reducing activity (TRA).

The current study provided longitudinal data on sexual stage immune responses with repeated assessments of (functional) sexual stage immune responses shortly after a single malaria episode. Most previous studies on naturally acquired immune responses to *P. falciparum* sexual stage antigens relied on cross-sectional study designs [17], [18], [23], [32], [33], [34], [35], [36], [37], making it difficult to determine factors associated with the induction or persistence of sexual stage immune responses. To the best of our knowledge, only three studies have previously determined the acquisition and loss of sexual stage immune responses in longitudinal settings. These studies collected samples at 3- or 6-monthly intervals [19], [24], [38], [39] and suggested that sexual stage antibody responses and functional TRA can be acquired rapidly [19] and may be more common in children than adults [23], [24], [39]. The current study focused on recently infected individuals and determined exposure to malaria parasites and immune responses in the weeks following a clinical episode, i.e. when there are potentially infectious gametocytes circulating against which transmission reducing immunity can act. Serological assessments were made at the time of first presentation with gametocytes and 4 and 12 weeks after enrolment, corresponding to 2–4 and 10–12 weeks after first presentation with gametocytes. None of the individuals in our cohort were re-infected with malaria parasites during 84-day follow-up. The prevalence and concentration of Pfs230 and Pfs48/45 antibodies were correlated with a longer duration of recent exposure to gametocytes. Less than 10% of the individuals who did not develop microscopically detectable gametocyte concentrations had antibodies against Pfs230 or Pfs48/45 one week after clearance of asexual parasitaemia. This proportion of seropositives showed a non-significant increase over time, which may be the result of immune-boosting by submicroscopic gametocyte densities that frequently accompany patent asexual parasite carriage and persist for several weeks [40], [41], [42] but were not measured in the current study. The use of molecular gametocyte detection methods, unavailable at the time, would have improved our study as some of the exposure to gametocyte antigens in our cohort will have been missed due to the limited sensitivity of microscopy for detecting low density gametocytaemia [9], [42], [43], [44], [45]. Submicroscopic infections were previously associated with the boosting of immune responses to schizont [46] and merozoite antigens [47] and it is therefore possible that sub-microscopic gametocyte densities may have had a similar immune-boosting effect in our study. Because only microscopy data were available, we were only able to test the association between exposure to relatively high, i.e. microscopically detectable, gametocyte densities and sexual stage immunity. There was a pronounced increase in sexual stage specific antibody prevalence and concentration in individuals who were exposed to microscopically detectable gametocyte densities during follow-up: more than one-third of individuals who carried gametocytes on at least two occasions during follow-up were positive for Pfs230 and Pfs48/45 antibodies four weeks after exposure to gametocytes. Previous studies often found no association between sexual stage immune responses and concurrent gametocyte prevalence or density [19], [23], [24], [39], although the association is both biologically plausible and supported by indirect evidence from literature. For example, a study using immunoprecipitation for immune responses to Pfs230 and Pfg 27/25 observed variation in the intensity of bands in relation to malaria exposure, suggestive of immune boosting during the malaria season [39]. Similarly, Pfs230 and Pfs48/45 antibody prevalence and concentration increased with increasing recent exposure to malaria infections in Indonesian migrants exposed to malaria in hyperendemic Papua [19].

Sexual stage immune responses also increased with age in our cohort of parasite carriers. This study was not specifically designed to examine age related acquisition of immune responses and the sample size is insufficient to disprove observations from previous studies that showed that sexual stage immune responses do not increase with age [23], [24], [39]. However, age may well be a modulating factor in the acquisition or maintenance of sexual stage immune responses after (recent) exposure to sexual stage antigens. The prevalence of antibodies against Pfs230 and Pfs48/45 was higher in adults where they also appeared to have a longer half-life. More stable sexual stage immune responses in adults were previously reported in the Gambia [38]. There may be an innate difference between children and adults in how acquired immune systems function [48], [49], resulting in more efficient immune response after recent exposure in adults compared to children [46], [50], [51]. In addition, higher cumulative exposure may have resulted in a more stable immune response in adults [38].

In line with other studies, total IgG antibody levels against MSP-1_19_ and AMA-1 showed a rapid decline over time after antigen exposure [52], [53], [54]. This probably reflects the lifespan of short-lived plasma cells after discontinued antigen exposure [51]. The rapid decline in sexual stage antibody titres after acute infection appears similar to that observed for antibodies to asexual stage antigens, suggesting that B cell responses may be regulated in a similar manner. Studies with much longer follow-up periods are needed to determine the longevity of antibody responses from long-lived plasma cells which may sustain low (but detectable) titres of antibodies for much longer periods. While the longevity of antibody responses is similar for sexual and asexual stages, our data nevertheless indicate clear differences in the kinetics of the two responses, consistent with differences in the timing of antigen exposure. Sexual stage immune responses increased in the first month of follow-up, following gametocyte exposure; asexual stage immune responses declined after asexual parasites were cleared and were not associated to gametocyte carriage.

We observed a strong association between the presence and concentration of sexual stage-specific antibody responses and functional TRA. Pfs230 antibody-related TRA involves complement-mediated lysis of gametes [55]; Pfs48/45 antibodies can inhibit zygote development independent of complement [56]. Serum samples with high reactivity to one or both of these antigens effectively reduced transmission. The observed association between Pfs230 and Pfs48/45 antibody prevalence and TRA was previously observed in field samples [23], [24], [34], [35] but the association was particularly strong in the current dataset: 11/12 samples that showed TRA≥90% were positive for both Pfs48/45 and Pfs230. The stronger association could be a consequence of using purified IgG rather than serum in the SMFA experiments, thereby reducing nonspecific effects of serum proteins. When we compared antibody concentration data with TRA, the association suggested a clear lower threshold of antibody concentrations above which high levels of TRA were assured. This resembles data from Pfs25/Pvs25 vaccine studies in human volunteers and animal models where a clear lower threshold could be defined above which all serum samples reduced transmission by>50% [4], [57]. The strength of the association could be partially explained by the study setting. We recruited individuals who were exposed to relatively low levels of malaria transmission and may therefore have less immunity to asexual antigens, which might cross-react with gametes, reduce their infectivity [58] and thereby confound the association between sexual stage immune responses and TRA. The association between sexual stage antibody responses and TRA may vary between settings [17], [18], [19], [23], [24], [32], [35], [59], [60]. In our study population, ∼25% of all participants showed reactivity to Pfs230 or Pfs48/45. This proportion is similar to that found in gametocyte carriers from Tanzania [23] and Cameroon [17] and Tanzanian adults exposed to intense transmission intensity [24] but twofold lower than that reported in Sri Lankan individuals exposed to low level transmission intensity [60]. This underlines the necessity of carrying out longitudinal studies in areas with different transmission characteristics to determine the generality of our findings and determine whether the strong associations that we report can be extrapolated to other regions.

In conclusion, our data indicate that antibody responses to Pfs230 and Pfs48/45 are boosted by recent exposure to gametocytes and that increased antibody levels are associated with the duration of carriage of gametocytes. Our predicted half lives of sexual stage antibodies of 70–90 days are in broad agreement with the predicted half life of gametocyte carriage of 55 days [40]. However, antibody levels are correlated with TRA and the low antibody titres observed in the latter stages of infection may be insufficient to affect gametocyte infectivity of mosquitoes.
